# Injectable Thermosensitive Hydrogel Containing Erlotinib‐Loaded Hollow Mesoporous Silica Nanoparticles as a Localized Drug Delivery System for NSCLC Therapy

**DOI:** 10.1002/advs.202001442

**Published:** 2020-11-03

**Authors:** Xiaohan Zhou, Xinlong He, Kun Shi, Liping Yuan, Yun Yang, Qingya Liu, Yang Ming, Cheng Yi, Zhiyong Qian

**Affiliations:** ^1^ Department of Medical Oncology State Key Laboratory of Biotherapy and Cancer Center West China Hospital Sichuan University, and Collaborative Innovation Center for Biotherapy Chengdu 610041 PR China

**Keywords:** erlotinib, localized drug delivery, non‐small cell lung cancer, sustained release, thermosensitive hydrogel

## Abstract

Erlotinib (ERT), oral administration agents, is one of the most pivotal targeted drugs in the treatment of non‐small cell lung cancer (NSCLC); however, its poor solubility, low oral bioavailability, and capricious toxicity limit broader clinical applications. In this paper, a novel injectable matrix is prepared based on hollow mesoporous silica nanoparticles (HMSNs) and thermosensitive poly(d,l‐lactide)‐poly(ethylene glycol)‐poly(d,l‐lactide) (PDLLA‐PEG‐PDLLA, PLEL) hydrogel to encapsulate and localize the sustained release of ERT for improved efficacy against NSCLC. The test‐tube‐inversion method shows that this ERT‐loaded hydrogel composite (ERT@HMSNs/gel) presents as an injectable flowing solution under room temperature and transfers into a physically crosslinked non‐flowing gel structure at physiological temperature.The ERT@HMSNs/gel composite shows a much longer intratumoral and peritumoral drug retention by in vivo imaging study. Notably, this injectable drug delivery system (DDS) provides an impressive balance between antitumor efficacy and systemic safety in a mice xenograft model. The novel ERT loaded HMSNs/gel system may be a promising candidate for the in situ treatment of NSCLC. Moreover, this study provides a prospective platform for the design and fabrication of a nano‐scaled delivery system for localized anticancer therapies.

## Graphical abstract

A novel injectable composite is developed based on hollow mesoporous silica nanoparticles (HMSNs) and thermosensitive hydrogelpoly(d,l‐lactide)‐poly(ethylene glycol)‐poly(d,l‐lactide) (PDLLA‐PEG‐PDLLA, PLEL) via mixing them in proportion at room temperature. The obtained in situ drug delivery platform (HMSNs/gel) can gelate under body temperature, and is employed for localized and sustained delivery of small molecule, erlotinib (ERT), to significantly promote its therapeutic efficacy and ameliorate drug‐related toxicity.

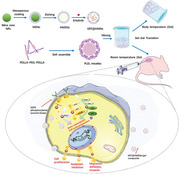

## 1 Introduction

Non‐small cell lung cancer (NSCLC) represents 80–85% of all lung malignant neoplasm and is one of the primary causes of cancer‐related mortality worldwide.^[^ ^1^, ^2^ ^]^ Epidermal growth factor receptor (EGFR) is overexpressed in several tumor types including NSCLC, and the activation of this protein is keenly associated with tumor cell proliferation, invasion, metastasis, angiogenesis, and apoptosis.^[^ ^3^, ^4^, ^5^ ^]^ Erlotinib (ERT), a selective and potent tyrosine kinase inhibitor (TKI), is effective and one of the frontline drugs for NSCLC therapy. It can bind to the ATP‐binding site of the tyrosine kinase domain of EGFR and block its auto phosphorylation, thereby inhibiting downstream signaling of the pathway in charge of cell proliferation, metastasis, and angiogenesis.^[^ ^6^ ^]^

At present, ERT is only available as a film‐coated tablet (Tarceva) in the market. Unfortunately, due to the low oral bioavailability and large absorption variation, in clinic Tarceva must be taken at a large daily dose (150 mg per day) relentlessly until disease progression and unacceptable toxicity occurs, which might be associated with high costs and risks. ERT has exhibited several dose‐related adverse effects, including skin rash, diarrhea, Stevens–Johnson syndrome, ocular lesions, and gastrointestinal perforations, some of which can be lethal for patients. From the patient compliance perspective, oral tablets administration is not easy for patients with gastrointestinal disorders or abnormalities.^[^ ^7^, ^8^ ^]^

Relentless efforts have been made in the field of nano‐technology‐based drug delivery systems (DDSs), which not only help to solve the fundamental shortcomings such as poor solubility, rapid degradation, and a transient biological activity, but also minimize the side effects via the selective accumulation at targeting tissues.^[^ ^9^, ^10^, ^11^ ^]^ Therefore, developing appropriate nano‐scaled drug carriers that could increase bioavailability and manifest expected efficacy at a low dosage would be a promising option to overcome the disadvantages of ERT mentioned above.

Indeed, ERT has been encapsulated into various nanocarriers, including poly‐lactic‐*co*‐glycolic acid (PLGA) nanoparticles,^[^ ^12^ ^]^ lipid–polymer hybrid nanoparticles,^[^ ^13^ ^]^ liposomes,^[^ ^14^ ^]^ albumin nanoparticles,^[^ ^15^, ^16^, ^17^ ^]^ and poly(*ε*‐caprolactone)‐poly(ethyleneglycol)‐poly(*ε*‐caprolactone) (PCEC) nanoparticles.^[^ ^18^ ^]^ These formulations have shown improved therapeutic efficacy and lessened drug‐related toxicities in different extent through intravenous administration. Still, the frequent injection was adopted to deliver and accumulate enough drugs at tumor site.

Predictably, ERT localized delivery based on a designed DDS can achieve similar anticancer effects via only one intratumoral injection which can minimize patients’ suffering and treatment cost. Among various DDS, implant hydrogel formulated antineoplastic agents exhibit as one of the most promising DDS for sustained release in cancer therapy.

The hydrogel is defined as 3D networks of crosslinked hydrophilic polymer chains.^[^ ^19^ ^]^ Given its unique structure, the hydrogel can absorb plenty of water or biological fluids, and simultaneously maintain their integrity. Because of high water content in the hydrogel, it can exhibit excellent biocompatibility and capability of encapsulating hydrophilic agents in therapeutic area.^[^ ^10^, ^20^ ^]^ Compared with the traditional preformed subcutaneous implantable hydrogels and other localized implantation materials, in‐situ‐forming injectable hydrogel systems have obtained great attention because of their noninvasiveness with the ability to carry therapeutic agents for site‐specific delivery, prolong drug action, improve patient compliance, and reduce systemic toxicity.^[^ ^21^, ^22^ ^]^

In our previous work, we have successfully synthesized a biodegradable and biocompatible thermosensitive hydrogel made of the amphiphilic triblock copolymer, poly(d,l‐lactide)‐poly(ethylene glycol)‐poly(d,l‐lactide) (PDLLA‐PEG‐PDLLA, PLEL), with the ability to self‐assemble into core–shell‐like micelles in water under room temperature and to transfer into physically crosslinked non‐flowing gel structure at physiological conditions.^[^ ^23^, ^24^, ^25^ ^]^

**Scheme 1:**
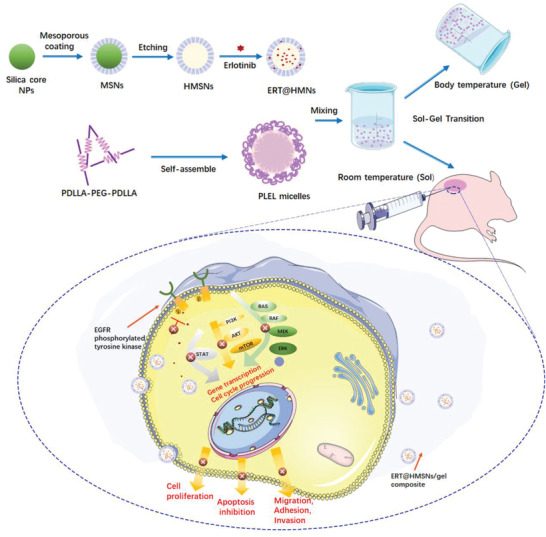
Schematic representation of the ERT@HMSNs and ERT@HMSNs/gel composite. HMSNs/gel in situ drug delivery platform was employed for localized and sustained delivery of small molecule, erlotinib, to promote its therapeutic efficacy and ameliorate drug‐related toxicity.

Due to the hydrophilic characteristics in the nature of hydrogels, the amount and homogeneity of ERT loaded into this high water content structure may be limited. Hence, in our study, hollow mesoporous silica nanoparticles (HMSNs) were adopted to encapsulate ERT for increasing drug loading and improving the solubility to solve the incompatibility of hydrophobic agents in hydrogel systems (**Scheme** **1**).

This novel HMSNs‐hydrogel matrix appears a homogeneous and stable solution by mixing ERT@HMSNs in PLEL hydrogel in proportion at room temperature Sequentially, the sharp sol–gel phase translation and sustained drug release behavior was evaluated by a series of assays both in vitro and in vivo minutely. We also investigated its possible application for the topical therapy of NSCLC in xenograft in nude mice.

## 2 Results and Discussion

### 2.1 Synthesis and Characterization of HMSNs and PELE Copolymer

The preparation route for HMSNs is shown in Scheme 1. Firstly, silica nanoparticles were synthesized based on a modified Stöber method. The mesoporous silica shell was then coated by a surfactant templating sol–gel approach on the silica core.

Na_2_CO_3_ aqueous solution was employed to remove the particle core to form the hollow core at a relatively mild temperature (50 °C). Finally, the surfactant hexadecyl trimethyl ammonium chloride (CTAC) was eliminated by HCl/ethanol mixture.

**Figure 1:**
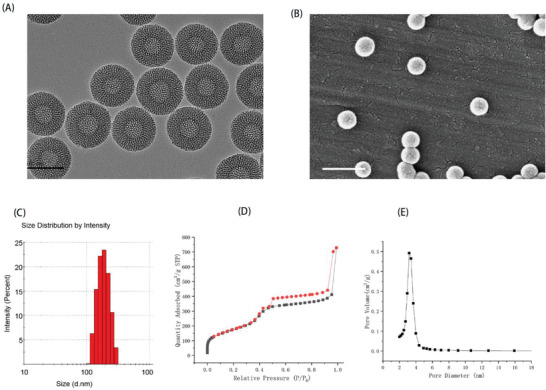
Characterization of resulting HMSNs. A) TEM image; B) SEM image; C) particle size distribution; D) nitrogen adsorption–desorption isotherm of HMSNs. Clearly, typical IV type adsorption curves were observed; E) pore size distribution. Scale bar: 200 nm (left) and 500 nm (right).

The morphology of resulting HMSNs was characterized by transmission electron microscopy (TEM) and scanning electron microscopy (SEM), both of which indicated that HMSNs were prepared successfully. According to **Figure** **1**A,B, the well‐formed spherical HMSNs had a smooth surface, uniform diameters of ≈150–200 nm, and a shell thickness of ≈30–40 nm. The particle size of the designed HMSNs was 175.17 ± 3.47 nm, with a polydispersity index (PDI) of 0.07 ± 0.03 (Figure 1C).

To confirm the specific surface area and porosity of resulting HMSNs, nitrogen adsorption–desorption isotherms with the corresponding Barrett–Joyner–Halenda (BJH) pore size distribution was measured. Nitrogen adsorption–desorption isotherms of obtained HMSNs displayed a typical IV isotherm, which exhibited open‐ended mesoporous feature,^[^ ^26^ ^]^ and the Brunauer–Emmett–Teller (BET) surface area was 642.03 m² g^−1^ with 1.13 cm^3^ g^−1^ pore volume (Figure 1D). The particle pore size around 3.2 nm was uniform, estimated by BJH method (Figure 1E).

The amphiphilic block copolymer, PDLLA‐PEG‐PDLLA copolymer, was successfully synthesized by ring‐opening polymerization. The Mn of the prepared copolymers was 4553 (PEG/PDLLA ratio is 1500:3053), which were calculated from the ^1^H nuclear magnetic resonance spectroscopy (^1^H NMR) spectrum (Figure S1, Supporting Information).

### 2.2 Preparation and Characterization of ERT@HMSNs and ERT@HMSNs/Gel Delivery System

ERT hydrophobic powder was successfully incorporated in HMSNs by using a solution‐solvent evaporation method. The drug loading (DL) capacity of the obtained ERT@HMSNs was 45.72 ± 0.14%, which was higher than traditional MSNs system mainly due to the large hollow interior drug storage volume. ERT can be more readily absorbed and concentrated into the hollow core and mesoporous shell of HMSNs.^[^ ^27^ ^]^

**Figure 2:**
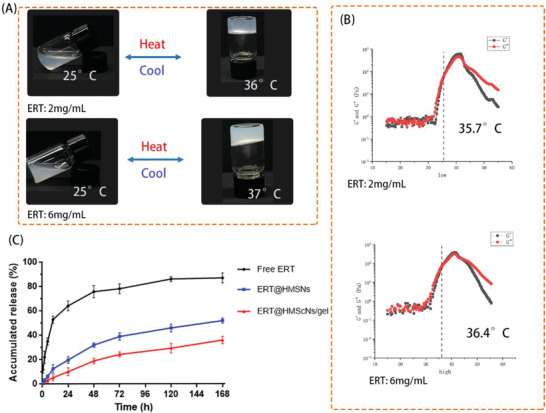
A) Reversible sol–gel phase transition of ERT@HMSNs/gel composite (ERT loading amount: 2 mg mL^−1^ and 6 mg mL^−1^) between 25 and 37 °C; B) Rheology analysis of ERT@HMSNs/hydrogel as a function of temperature; C) drug release profiles in vitro.

PLEL hydrogel based on a central PEG block and end PDLLA blocks exhibited a reversible temperature‐dependent sol–gel–sol transition, which is associated with micelles aggregation.^[^ ^23^ ^]^ It was reported previously that the micelles made of amphiphilic copolymers tend to be driven by the interaction of their hydrophobic core and at higher temperatures form physical crosslinked points which result in micelle crosslinked networks.^[^ ^28^ ^]^ In this investigation, ERT@HMSNs solution could be easily incorporated into the PLEL hydrogel via simple mixture in appropriate ratio under room temperature, and thus a homogeneous and free‐flowing HMSNs/PLEL injectable hydrogel loading ERT was obtained. As shown in **Figure** **2**A, the resulting hydrogel composites underwent sol and gel physical states as temperature went up. Both HMSNs/gel samples, containing ERT of 2 mg mL^−1^ and 6 mg mL^−1^, could maintain no‐flow condition at least 1 min at 37 °C, implying the gelation behavior at body temperature.

Simultaneously, the thermosensitive transition of hydrogel samples was also investigated through rheological measurements. Detailly, both storage modulus (G′) and loss modulus (G”) of HMSNs/gel composites were low independent of temperature ranging from 4 to 30 °C, indicating the well flowability and injectability without the risk of syringe clogging upon injection. As temperature increased around body temperature, G’ and G” raised abruptly corresponding to the sol–gel transition. The gelation temperature (Tgel) was noted as the temperature when G’ and the storage modulus (G”) met with each other,^[^ ^29^ ^]^ because G” is an elastic component, and G” is a viscous component of the complex modulus. From a mechanical point of view, a gel region can be defined by the zone where the elastic component (G’) overwhelms the viscous component (G”).

Specially, a high concentration of ERT@HMSNs led to a slight increase of Tgel compared with that of low concentration (35.7 and 36.4 °C, respectively). A possible explanation for this phenomenon is that HMSNs disturbed the original compact arrangement of PLEL micelles. The sol–gel transition feature demonstrated further clinical application of HMSNs/gel delivery system as an in situ gel‐forming controlled drug‐delivery carriers.

In the setting of cancer therapeutics, the sustained and controlled drug release is a basic property for future clinical applications. Based on pharmacology theory, fast release behavior is associated with the drug diffusion in plasma rather than the concentration at the targeted foci, thus increasing the invalid release and off‐target toxicity.^[^ ^30^ ^]^ The cumulative release curve was displayed (Figure 2C). A persistent release profile of ERT was observed. During the first 72 h, 39% and 24% of ERT released were recorded in HMSNs and HMSNs/gel groups, respectively, which were significantly lower than that of free ERT group. From these release curve, we could draw a conclusion that the 3D structure of hydrogel was able to slow down the premature drug release and thus increased the drug accumulation at the tumor lesions.

The in vitro storage stability of different ERT loaded formulations were evaluated under general storage condition (4 °C). Figure S2, Supporting Information displayed the gross views of the ERT@HMSNs and ERT@HMSNs/gels at predetermined time points. At 4th and 7th day, ERT powder diffused out of HMSNs and HMSNs/gel, respectively, which indicated that hydrogel could slightly improve the storage stability of formulations

### 2.3 Cellular Uptake and Cytotoxicity Assays In Vitro

**Figure 3:**
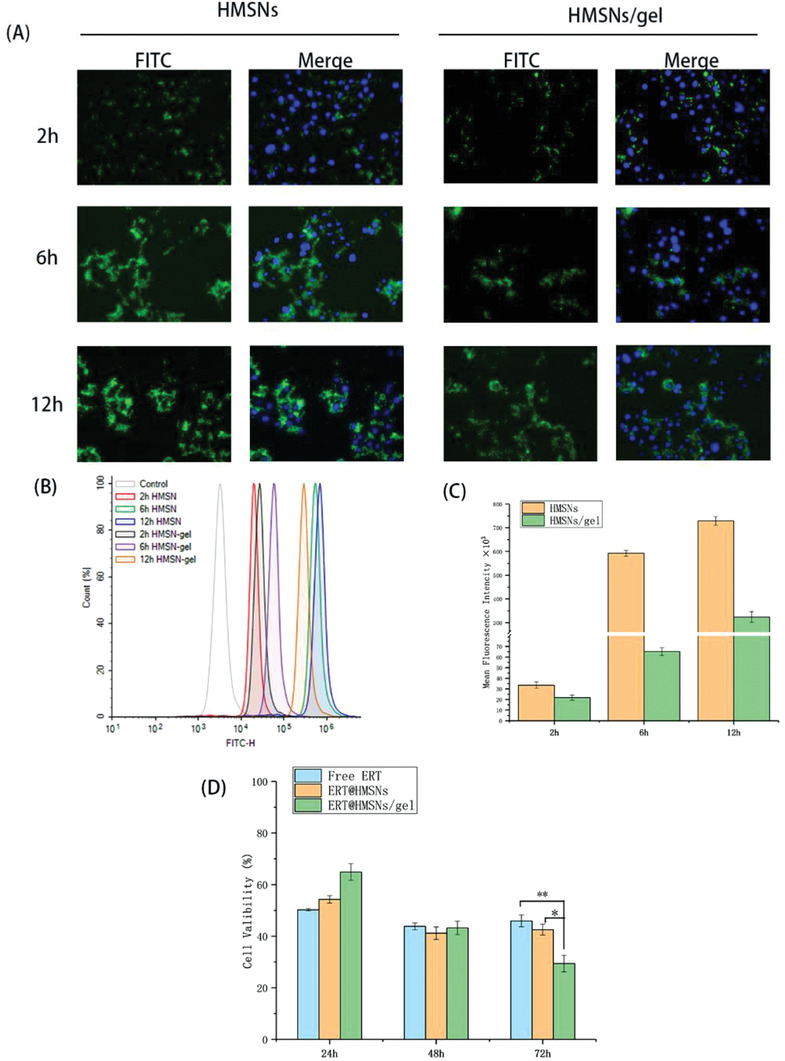
Cellular uptake of different ERT formulations in A549 cells. The FITC‐labeled formulations were added into transwell inserts and then incubated with cells at different time. A) HMSNs accumulation was represented by the fluorescence of FITC which was observed by fluorescence microscopy. B,C) Quantitative analysis of fluorescent intensity of FITC was determined by flow cytometry; D) Viability of A549 cells incubated with ERT@HMSNs and ERT@HMSNs/gel in Transwell co‐culture systems for 24, 48, and 72 h. All quantitative data are given as mean ± SD (*n* = 3). “*” and “**” mean *p* < 0.05 and 0.01, respectively. Scale bar: 20 µm

Efficient uptake and internalization of drug‐loaded nanoparticles are essential prerequisites for their therapeutic efficacy. To investigate the transport ability of ERT@HMSNs in vitro, the fluorescein isothiocyanate (FITC)‐labeled HMSNs, fluorescence microscope, and flow cytometer were adopted. We have chosen the most representative images based on cell density and fluorescence degrees. The fluorescence signals and intensity of FITC in NSCLC cells were shown in **Figure** **3**A–C. The results demonstrated that within the initial 2 h, some similar green fluorescent signals were observed in both groups, indicating HMSNs could quickly release from the hydrogel matrix at the very beginning of treatment.

This phenomenon named initial burst release was also observed in our previous study, which can contribute to rapid onset of antitumor agents via increasing drug concentration in tumor lesions.^[^ ^24^ ^]^

Regarding HMSNs groups, after 6 h, green fluorescence became much stronger, implying HMSNs could be pumped into cytoplasm, helping ERT effectively play antitumor effects. Given the similar fluorescence signal after 12 h, we could draw a conclusion that the uptake of HMSNs solution nearly reached saturation point within 6 h. The fluorescence intensity of HMSNs/gel group were slowly enhanced as time went on, indicating that HMSNs could persistently release from gel matrix and pump into cytoplasm.

Cytotoxicity assay provides crucial information about the therapeutic potential of ERT and whether the released ERT from delivery vectors was still pharmacologically active. 3‐(4,5‐Dimethyl‐2‐thiazolyl)‐2,5‐diphenyl‐2‐H‐tetrazolium bromide (methyl thiazolyltetrazolium, MTT) assay was performed on A549 cells. A Transwell co‐culture system was employed to create a drug depot, and the initial medium was replaced by fresh medium every 24 h to mimic the drug clearance in vivo. The excellent cytocompatibility of blank PLEL hydrogel and HMSNs have been investigated and verified in our previous studies.^[^ ^23^, ^31^ ^]^ Figure 3D illustrated the viability of A549 cells after 24, 48, and 72 h incubation with free ERT, ERT@HMSNs, and ERT@HMSNs/gel. During the first 24 h of incubation, the drug‐loaded hydrogel matrix showed a lower cell proliferation inhibition compared with free ERT. This might be attributed to the delayed release of the drug from the micelle networks. After replacing initial medium, the viability of cells in free ERT and ERT@HMSNs solution decreased much slowly upon time. By contrast, the viability of cells treated with ERT@HMSNs/gel showed a negative correlation with time, indicating the sustained release of drugs from micellar networks. Moreover, compared between free ERT and ERT@HMSNs groups, encapsulated ERT in HMSNs could slightly enhance the cytotoxic activity after 24 h, however, no significant differences were observed between these groups. This might be ascribed to ERT slow release from nanoparticles.^[^ ^32^ ^]^

### 2.4 Biodistribution and Retention Studies

In vivo imaging study was conducted to examine the fate of different ERT formulations. To track the lipophilic drugs in vivo, a near infrared ray (NIR) dye, fluorescent probe 1,1′‐dioctadecyl‐3,3,3′,3′‐tetramethylindotricarbocyanine iodide (DiR), was applied as a representative of ERT.

**Figure 4:**
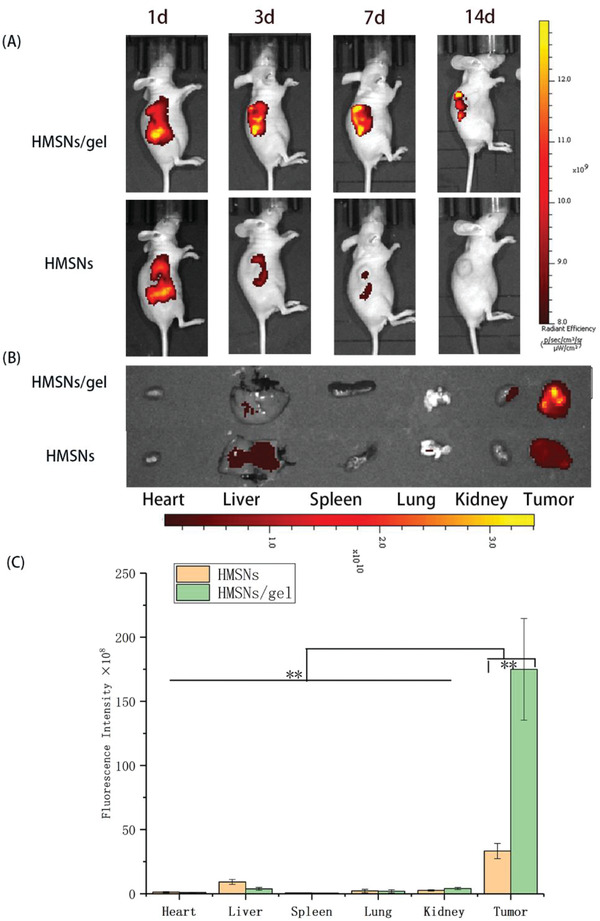
In vivo biodistribution and retention studies by NIR imaging. A) The NIR real‐time images of A549 xenograft models after i.t. injection of DiR@HMSNs formulation and DiR@HMSNs/hydrogel composite at 1st, 3rd, 7th, and 14th days B) with the NIR images of ex vivo tumors and mean organs on 14th day after the initial injection; C) Quantitative analysis was conducted to determine the fluorescence in ex vivo mean organs and tumors. All quantitative data are given as mean ± SD (*n* = 3). “**” means *p* < 0.01.

As is illustrated in **Figure** **4**, both groups exhibited that the NIR signals slowly decayed over time. Following peritumoral and intratumoral injection, the fluorescence signals in tumor site of DiR@HMSNs/gel group were much stronger at the same time point and decayed much slower compared with that of DiR@HMSNs group.

Furthermore, to evaluate the drug residue in mean organs, the mice were sacrificed, and quantitative analysis of fluorescence intensity in ex vivo mean organs and tumors was conducted. As expected, the fluorescence intensity of hydrogel group was much stronger in tumor and weaker in normal organs than that of HMSNs group (Figure 4B,C).

Notably, no obvious DiR fluorescent signals were observed in heart, spleen, and lung in both groups, however, weak fluorescent signals were found in liver and kidney, which might be the excretion of kidney and the highly macrophage uptake of liver.^[^ ^33^ ^]^

From these results, it could be deduced that the persistent and local release ERT from HMSNs/gel formulation led to preferential drug accumulation in tumor lesions, rather than exposure into systemic circulation, during which drug‐related toxicity would be ameliorated.

### 2.5 Antitumor Efficiency In Vivo

**Figure 5:**
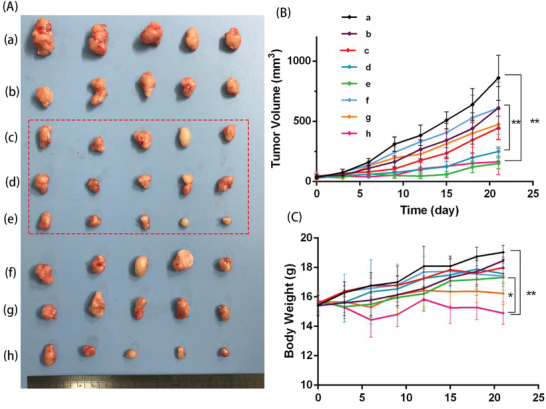
In vivo antitumor efficiency of different ERT formulations on NSCLC xenograft models. A) Photographs of subcutaneous tumors in each group: a) NS; b)ERT@HMSNs (i.t. 50 mg kg^−1^); c) ERT@HMSNs/gel (i.t. 25 mg kg^−1^); d) ERT@HMSNs/gel (i.t. 50 mg kg^−1^); e) ERT@HMSNs/hydrogel (i.t. 100 mg kg^−1^); f) Tarceva (p.o. 25 mg kg^−1^ per day); g) Tarceva (p.o. 50 mg kg^−1^ per day); h) Tarceva (p.o. 100 mg kg^−1^ per day). B) The tumor growth curves of each group. C) Body weight changes of mice as a function of time in each group. The dashed ellipse emphasized groups given ERT@HMSNs/gel with different drug concentration. All quantitative data are given as mean ± SD (*n* = 5). “*” and “**” mean *p* < 0.05 and 0.01, respectively.

The efficient cellular uptake in vitro and higher local retention in vivo could offer a theoretical basis of greater antitumor efficacy of ERT in HMSNs/PLEL gel DDS. To further evaluate the in vivo antitumor ability, the nude mice xenograft model of NSCLC was treated with different ERT formulations and Tarceva. According to the tumor image (**Figure** **5**A) and tumor growth curves (Figure 5B), oral Tarceva, local injectable ERT@HMSNs and ERT@HMSNs/ gel all exhibited efficient inhibition to the development of A549 tumor. The tumor volume in ERT@HMSNs/PLEL gel group (i.t. 100 mg kg^−1^) was as small as that in Tarceva (p.o. 100 mg kg^−1^ per day) group. Obviously, adopting different drug formulation and administration route could significantly elevate the therapeutic effects of ERT and reduce dosage. Compared to ERT@HMSNs, ERT@HMSNs/gel (i.t. 50 mg kg^−1^) could further significantly enhance the antitumor efficacy with a tumor volume percentage of 30.3% on the 21st day (*p* < 0.01), which mainly attributed to the sustained agent release from hydrogel and the excellent intratumoral and peritumoral ERT accumulation. Hence, optimized delivery system of ERT could considerably elevate the therapeutic effects with a relatively low dosage. Additionally, for gel groups, when the dosage of ERT in HMSNs/hydrogel system rose to 50 mg kg^−1^ or 100 mg kg^−1^, the rapid growth was almost completely suppressed, which indicated that ERT in gel DDS could improve the therapeutic effects in a dose‐dependent manner.

**Figure 6:**
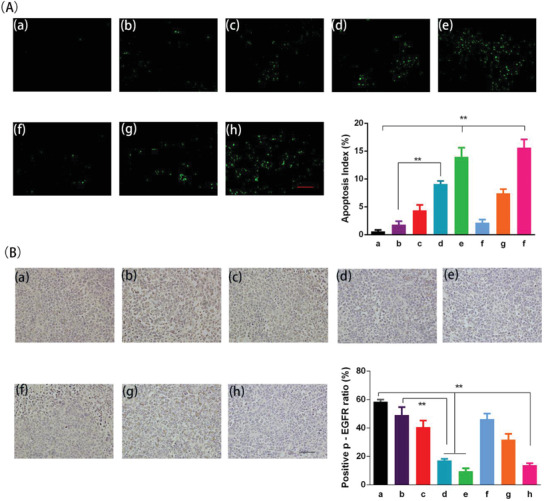
Apoptosis (A) and p‐EGFR level (B) of A549 tumors in different treatment groups. a) NS; b) ERT@HMSNs (i.t. 50 mg kg^−1^); c) ERT@HMSNs/gel (i.t. 25 mg kg^−1^); d) ERT@HMSNs/gel (i.t. 50 mg kg^−1^); e) ERT@HMSNs/hydrogel (i.t. 100 mg kg^−1^); f) Tarceva (p.o. 25 mg kg^−1^ per day); g) Tarceva (p.o. 50 mg kg^−1^ per day); h) Tarceva (p.o. 100 mg kg^−1^ per day). Scale bar: 100 µm. All quantitative data are given as mean ± SD (*n* = 5). “**” means *p* < 0.01.

Moreover, intracellular markers of apoptosis and phosphorylation in tumor tissues of model mice were analyzed by terminal deoxynucleotidyl transferase‐mediated nick‐end labeling (TUNEL) and p‐EGFR staining. The expression of TUNEL implied more pronounced apoptosis of tumor cells in the ERT@HMSNs/gel (i.t. 100 mg kg^−1^) and Tarceva (p.o. 100 mg kg^−1^ per day) groups (apoptotic rate 14.1% and 15.1%, respectively) (**Figure** **6**A). In previous studies, ERT was proved to target the EGFR tyrosine kinase domain through reversibly binding to the ATP‐binding site of the kinase. The interaction could prevent the phosphorylation of EGFR and the subsequent signal transduction, thus leading to tumor cell apoptosis and reducing cellular proliferation. The results from our texts in vivo also correspond with this theory. Different formulated‐ERT could downregulate the levels of p‐EGFR in tumor tissues (Figure 6B). The downregulation effects of ERT@HMSNs/gel are significantly stronger than that of ERT@HMSNs at the same drug dosage (i.t. 50 mg kg^−1^). (17.2% and 49.3%, respectively) (*p* < 0.01), which corresponds to the better antitumor efficacy observed in the pharmacodynamic results.

### 2.6 Side Effects Evaluation

**Figure 7:**
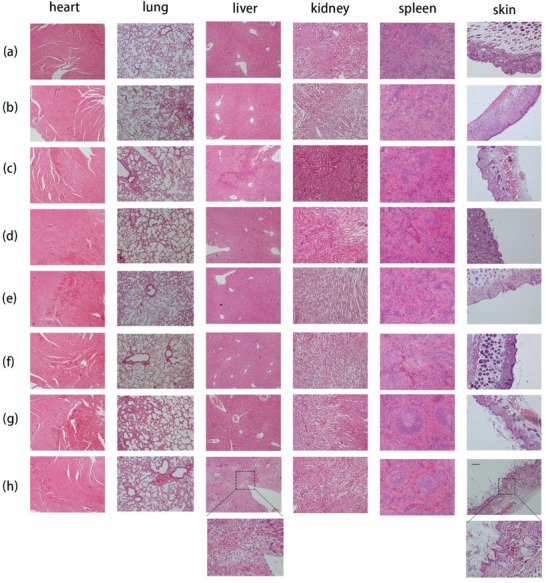
The H&E sections of hearts, livers, spleens, lungs, kidneys, and skins after different administration in A549 model. Each group (*n* = 3) was a) NS; b)ERT@HMSNs (i.t. 50 mg kg^−1^); c) ERT@HMSNs/gel (i.t. 25 mg kg^−1^); d) ERT@HMSNs/gel (i.t. 50 mg kg^−1^); e) ERT@HMSNs/hydrogel (i.t. 100 mg kg^−1^); f) Tarceva (p.o. 25 mg kg^−1^ per day); g) Tarceva (p.o. 50 mg kg^−1^ per day); h) Tarceva (p.o. 100 mg kg^−1^ per day). Scale bar: 50 µm.

In the space of this experiment period, treatment‐related death was not recorded. Compared to the control group, ERT@HMSNs‐ and ERT@HMSNs/gel‐treated animals presented no significant alterations in body weight, activity, or clinic signs, suggesting both HMSNs and hydrogel are safe and promising candidates for intratumoral usage. While it was observed that oral administration of Tarceva with a high daily dose significantly decreased the body weight of experimental mice throughout the course the treatment compared with high dose hydrogel group (*p* < 0.05) (Figure 5C). Localized injection of ERT@HMSNs/gel prevented the weight reduction, since the systemic toxicity caused by the high concentration of ERT in plasma was minimized. Part of mice, given Tarceva 100 mg kg^−1^ per day, suffered treatment‐related macroscopic changes in the skin (ulcers and desquamation) (Figure S3, Supporting Information), which might be stemming from the high‐level expression of EGFR in the skin. These lesions, however, were transient and dissipated with continued treatment. Similarly, the hematoxylin and eosin staining (H&E) results suggested that high dosage of Tarceva caused organic disorder in the liver, including hydropic degeneration in hepatic lobules. Histologically, the skin lesions consisted of diffuse, mild to moderate epidermal acanthosis, epidermal hyperkeratosis, and focal escharosis (**Figure** **7**).

## 3 Conclusion

We have successfully developed an injectable nano‐scaled DDS, with thermosensitive hydrogels incorporated ERT‐loaded HMSNs. This novel system showed its therapeutic potential in vitro and in vivo. Concomitantly, a convenient approach to preparing ERT@ HMSNs/gel composite system was shown.

The ERT@ HMSNs loaded hydrogel composite exhibited a stable sol–gel transition from room temperature to a physiological temperature. The in vitro release profiles and in vivo distribution both indicated that this unique DDS could form a depot under body conditions and achieve a preferential accumulation of drug at the site of administration. In vivo antitumor experiments showed that the ERT@ HMSNs/gel groups could produce better treatment effects with no apparent changes in body weight and main organs.

Overall, it can be safely concluded that the ERT@ HMSNs/gel is a promising DDS for in situ administration in NSCLC treatments, although storage stability needs to be optimized further. This designed injectable biodegradable DDS may allow a much lower dosage regimen without the loss of therapeutic effect compared with current commercial tablets Tarceva in the market for NSCLC treatment, and thus could be potentially applied in combination therapies for NSCLC in future.

## 4 Experimental Section

##### Materials

Tetraethyl orthosilicate (TEOS), tetraethylammonium hydroxide (TEAH), CTAC, PEG (Mn = 1500), stannous octoate (Sn(Oct)2, 95%), MTT, and 4′,6‐diamidino‐2‐phenylindol (DAPI) were obtained from Sigma‐Aldrich (Saint Louis, USA), d,l‐Lactide (d,l‐LA) and ERT were bought from Meilun Co., Ltd (Dalian, China). FITC, DiR were supplied from the Beijing Fanbo Biochemicals Company (Beijing, China). A549 cells were purchased from the American Type Culture Collection (ATCC, Rockville, MD), which grew in Dulbecco's modified Eagle's medium (DMEM) supplement with 10% fetal bovine serum (FBS) and 1% penicillin/streptomycin (Gibco, USA). The cells were maintained in a 37 °C incubator with a humidified 5% CO_2_ atmosphere.

All the materials used in this article were analytic reagent grade and used as received without further purification.

##### Synthesis and Characterization of HMSNs and PLEL Triblock Copolymer

Typically, silica seeds were first synthesized by the Stöber method.^[^ ^34^ ^]^ In brief, 0.4 mL aqueous ammonia (30–33 wt%,) and 1.6 mL deionized water were mixed with absolute ethanol (9.6 mL) stirring for 30 min, and then the mixture of TEOS (0.12 mL) and ethanol (10 mL) was added. After stirring for 24 h at room temperature, the seeds were obtained. Secondly, 25% CTAC and 0.02 g TEAH were added into the seeds solution and the mixture was stirred for 1 h at the temperature of 80 °C. After that 0.2 mL TEOS solution was dropped at a certain speed. The system was reacted overnight and the core–shell silica nanoparticles were received. Subsequently, a selective etching approach was employed to produce HMSNs. The as‐prepared core–shell silica nanoparticles were centrifuged and re‐dispersed in Na_2_CO_3_ aqueous solution. The mixture was stirred at 80 °C for 2 h to remove the solid silica core, and then rinsed with concentrated HCl/ethanol (v/v = 1:100) and deionized water three times for the removal of CTAC micelles residual in the mesoporous shell.

The particle size distribution was determined by a Malvern Nano‐ZS 90 laser particle size analyzer (Worcestershire, UK). TEM, (H‐6009IV, Hitachi, Japan) and SEM (JSM‐7500F, JEOL, Japan) were used to examine the morphology and shape of HMSNs. Nitrogen absorption and desorption isotherms of resulting HMSN sample was acquired by an ASAP 2460 (Micromeritics, USA) nitrogen adsorption apparatus. The BET and BJH measurement were selected to calculate the specific surface area and corresponding pore characteristics, including pore size and pore volume. The sample was degassed at 120 °C for 24 h under nitrogen atmosphere prior to the measurement.

Amphiphilic block copolymers, PLEL were synthesized from the methoxypolyethylene glycols (mPEG) and d,l‐lactide (LA) by the ring‐open polymerization according to the previous works.^[^ ^23^, ^25^ ^]^ The obtained PLEL copolymers were characterized by ^1^H NMR (Varian 400 spectrometer, Varian, USA).

##### Preparation and Characterization of the ERT‐Loaded HMSNs (ERT@HMSNs)

ERT@HMSNs were prepared by a solution–solvent evaporation approach. In brief, ERT was first dissolved in methanol solution and then 5 mL of the as‐prepared ERT solution was added into 5 mL HMSNs suspension. After string overnight under room temperature, the mixture was evaporated under vacuum conditions with the sonicate until the total volume was less than 3 mL. Then the remaining solution was centrifuged and washed, and finally degassed for 2 h at a suitable temperature to remove solvent and crystalize drug in the hollow cavity.

The drug loading capacity were assessed by high performance liquid chromatography HPLC instrument (HPLC 1260, Agilent, US) with a C^18^ column (4.6 mm × 150 mm × 5 µm, Grace Analysis column). The mobile phase solvent consisting of acetonitrile, water, and trifluoroacetic acid (40/60/0.1, v/v) was pumped at a flow rate of 1.0 mL min^−1^. Detection was taken on a diode array detector (1260 DAD VL) at a wavelength of 331 nm. Results were calculated according to the following equations: DL% = Amount of drug / Amount of HMSN + drug 100%.^[^ ^35^ ^]^

##### Preparation and Characterization of ERT@HMSNs/Hydrogel

ERT@HMSNs/hydrogel was prepared by two steps. First, PLEL copolymer was dissolved in PBS at room temperature and cooled to 4 °C. Secondly, the pre‐made ERT@HMSNs sol was mixed with PLEL sol in the right proportion to form homogeneous solution, the concentration of PLEL copolymer adjusted to 15 wt%.

The thermosensitive sol–gel phase transition behavior of ERT HMSNs hydrogel composite was observed by the test‐tube‐inverting method with a 3 mL tightly screw‐capped vial.^[^ ^36^ ^]^ Briefly, 1 mL drug loaded hydrogel samples, containing 2 or 6 mg mL^−1^ ERT were added to vials and were heated at a rate of 1 °C min^−1^, from 10 °C to the temperature when precipitation occurred. At each temperature point, the sample was equilibrated for 10 min, and the “gel formation” was defined when no significant flow was observed after tilting the vials within 1 min.

The dynamic rheological measurements were carried out to further explore the influence of ERT concentration on thermosensitive sol–gel–sol phase transition of HMSNs/PLEL system by the HAAKE Rheostress 6000 rheometer (Thermo Scientific, USA). The cooled samples were placed between parallel plates with a diameter of 20 mm and with a gap of 1 mm. In order to minimize solvent evaporation, samples were carefully covered with a thin layer of low‐viscosity silicone oil. The temperature was controlled at a heating rate of 1 °C min^−1^ with a temperature precision of ±0.1 °C. The G′ and G” were measured under controlled stress of 4.0 dyn cm^−2^ and a frequency of 1.0 Hz. The temperature of gelation was observed through changes of the modulus.

The release behaviors of ERT from ERT@HMSNs and ERT@HMSNs/gel groups were determined by a modified dialysis method in vitro. 1 mL formulations, containing 1 mg ERT, were placed in dialysis bags. Then these dialysis bags were incubated in 10 mL of PBS (pH 7.4) with Tween80 (0.5 wt%) at 37 °C with gentle shaking (100 rpm). The incubation medium was entirely replaced by the same volume of fresh pre‐heated medium PBS at predetermined time points, and all the collected PBS was stored at −4 °C before further HPLC analysis. All results were the mean of three individual tests.

Concomitantly, the influence of different formulation and ERT concentration on storage stability was determined. Different ERT loaded formulations were stored at 4 °C, and the changes were observed at predetermined time points.

##### Cellular Uptake and Cytotoxicity Assays In Vitro

HMSNs were labeled with FITC by the following procedure. Amine group modified HMSNs were synthesized through adding aminopropyltriethoxysilane (APTES) together with TEOS during the core–shell NPs formed and treated with the similar procedures mentioned above. Then, 20 mg amine group modified HMSNs were dispersed into ethanol containing 0.4 mg FITC and reacted for 12 h under dark shaking conditions at room temperature.^[^ ^37^ ^]^ The product was rinsed with both deionized water and ethanol each for three times to remove free FITC.

The effective cellular uptake of the drug‐loaded HMSNs released from PLEL hydrogel composite was evaluated through the 24‐well Transwell (Corning) co‐culture system. NSCLC cells, A549, were seeded in 24‐well Transwell (40 000 cells per well) with 500 µL DMEM medium containing 10% FBS and penicillin–streptomycin liquid at 37 °C for 24 h, to bring the cell well dispersed. Then 100 µL PBS (control group), FITC‐labeled HMSNs, and FITC‐HMSNs/gel composite were directly added to the upper inserts of the Transwell, and mildly heated for the gelation of PLEL solution. Subsequently, these FITC containing inserts were put into the cells cultured plate to incubate with A549 for 2, 6, 12 h at 37 °C. The treated cells were observed by fluorescence microscopy (Olympus, China) after being washed three times with PBS, fixed with a 4% paraformaldehyde solution for 30 min and stained with DAPI. Also, the intensity of FITC was analyzed by flow cytometry (Calibur, BD, USA).

The cytotoxicity of ERT‐loaded formulations in the study on A549 cells was evaluated by the MTT assay and Transwell (Corning) co‐culture system. A549 cells were seeded in 24‐well culture plates at a density of 2 × 10^4^ cells per well and cultured for 24 h at 37 °C, and then treated with 100 µL PBS (control group), free ERT solution, ERT@HMSNs, and ERT@ HMSNs/gel (ERT, 400 µg mL^−1^) as described in the previous section. To imitate the drug clearance in vivo, the initial medium in all wells was replaced by fresh medium every 24 h. After co‐cultivation for 24, 48, or 72 h, the cells were subjected to MTT assay, and the relative viability was expressed as a percentage compared to the control group.^[^ ^38^ ^]^

##### Animals and NSCLC Xenograft Model

Female balb/c nude mice (16–18 g) were purchased from HFK Bioscience Co., Ltd (Beijing, China). The mice were housed in an SPF environment with free access to food and water. All animal experiments were approved by the Animal Care and Use Committee of Sichuan University (Chengdu, China). Tumor‐bearing mice model was established by subcutaneously injecting 5  ×  10^6^ A549 cells in the right flank regions. When the tumor volume approximately reached 50 mm^3^, the treatment was initiated.

##### Biodistribution and Retention Studies

A hydrophobic near infrared dye, DiR was used as a model drug and the localized retention capacity of the different DDS over the whole body was monitored by an in vivo fluorescence imaging system. The tumor‐bearing mice were randomly divided into two groups and injected with 200 µL of DiR@HMSNs (DiR, 20 µg mL^−1^) and DiR@HMSNs/gel intratumorally or peritumorally. Then they were imaged via an IVIS imaging system (Perkin Elmer, USA) at 1st, 3rd, 7th, and 14th day. On 14 days post‐injection, the mice were sacrificed, and the heart, liver, spleen, lung, kidney, and tumor were harvested to evaluate the ex vivo organs’ fluorescence intensity. Concomitantly, the quantitative fluorescent intensity was acquired by using the onboard software.

##### Treat Plan and Antitumor Efficiency In Vivo

When tumor volumes reached 50 mm^3^ or so, mice were randomly divided into eight groups (*n*  =  5). Each group of mice was treated with NS (control group); ERT@HMSNs (i.t. 50 mg kg^−1^); ERT@HMSNs/hydrogel (i.t. 25 mg kg^−1^, 50 mg kg^−1^, and 100 mg kg^−1^, respectively); Tarceva (p.o. 25 mg kg^−1^, 50 mg kg^−1^, and 100 mg kg^−1^ per day, respectively). Except for Tarceva, treatments were administered through intratumoral or peritumoral injection with 200 µL of formulations above mentioned by a single injection. Commercial tablets of ERT, Tarceva, were used as positive control with rational dosage referring to previous reports.^[^ ^39^ ^]^

The tumor size was measured by a vernier caliper every 3 days and calculated by the equation: *V* = *A* × *B* ^2^/2, where *A* is the long axis and *B* is the short axis. The body weight was recorded every 3 days also.

On the 21st day of treatment, the mice were sacrificed. Tumors and main visceral organs (heart, liver, spleen, lung, and kidney) and skin were washed with PBS after separating, and then preserved in 4% paraformaldehyde for the following immunofluorescent and immunohistochemical analysis.^[^ ^40^ ^]^

##### Side Effects Evaluation

All mice were continuously observed after administration, including the general conditions (the skin, activity, behavior, energy, secretion, and other clinic signs), body weight, and mortality. After sacrificing, the harvested hearts, livers, spleens, lungs, kidneys, and skins were used to assess histological changes using H&E and evaluated under microscope (Biological microscope, BX53, Olympus Corp, Japan).

##### Immunohistochemistry Analysis of TUNEL and p‐EGFR

Apoptosis of tumor cells was evaluated by TUNEL staining assay. After being fixed by 4% paraformaldehyde, the tissues were embedded in paraffin and sectioned.^[^ ^38^ ^]^ An in situ cell death detection kit (DeadEnd Fluorometric TUNEL System, Promega, Madison, USA) was used for TUNEL staining. The staining was carried out according to the manufacturer's protocol. Tumor tissue sections were prepared as described above. The expression of p‐EGFR in tumor tissues was detected and evaluated via immunohistochemistry (IHC) staining and biotinylated rabbit anti‐human p‐EGFR antibodies.^[^ ^41^, ^42^ ^]^ The samples were rehydrated through a graded alcohol series before embedded with paraffin. After IHC staining, tumor sections were counterstained with hematoxylin solution for nuclei coloration and the finished sections were observed under microscopy. In tumor tissue sections, five equal‐sized fields at 800 magnification were randomly chosen and analyzed. The apoptosis rate and p‐EGFR labeling indexes were calculated as number of apoptosis and p‐EGFR positive cells/total number of cells counted in five randomly selected fields by two independent investigators in a blinded fashion.^[^ ^43^ ^]^

##### Statistical Analyses

All the data were expressed as mean ± standard deviation (SD). One‐way analysis of variance (ANOVA) and Student's *t*‐test were used for statistical analysis. *p* < 0.05 or *p* < 0.01 marked with “*” or “**” indicated that the results were statistically significant. The statistical analyses were carried out via SPSS 17.0 software (Chicago, IL, USA).

## Conflict of Interest

The authors declare no conflict of interest.

## Supporting information

Supporting information
