# Supplementary material for: Injectable Thermosensitive Hydrogel Containing Erlotinib‐Loaded Hollow Mesoporous Silica Nanoparticles as a Localized Drug Delivery System for NSCLC Therapy
Source: Adv Sci (Weinh). 2020 Nov 3;7(23):2001442. doi: 10.1002/advs.202001442 (PMC7709975; doi:10.1002/advs.202001442)
Supplement: Supplementary file 1 — Supporting information [file ADVS-7-2001442-s001.pdf]

WILEY-VCH

© 2020 Wiley-VCH GmbH

# Supporting Information

for *Adv. Sci.*, DOI: 10.1002/advs.202001442

# Injectable Thermosensitive Hydrogel containing Erlotinib-loaded Hollow Mesoporous Silica Nanoparticles as Localized Drug Delivery System for NSCLC Therapy

*Xiaohan Zhou, Xinlong He, Kun Shi, Liping Yuan, Yun Yang, Qingya Liu, Yang Ming, Cheng Yi, Zhiyong Qian**

Supporting Information

# Injectable Thermosensitive Hydrogel containing Erlotinib-loaded Hollow Mesoporous Silica Nanoparticles as Localized Drug Delivery System for NSCLC Therapy

*Xiaohan Zhou, Xinlong He, Kun Shi, Liping Yuan, Yun Yang, Qingya Liu, Yang Ming, Cheng Yi, Zhiyong Qian**

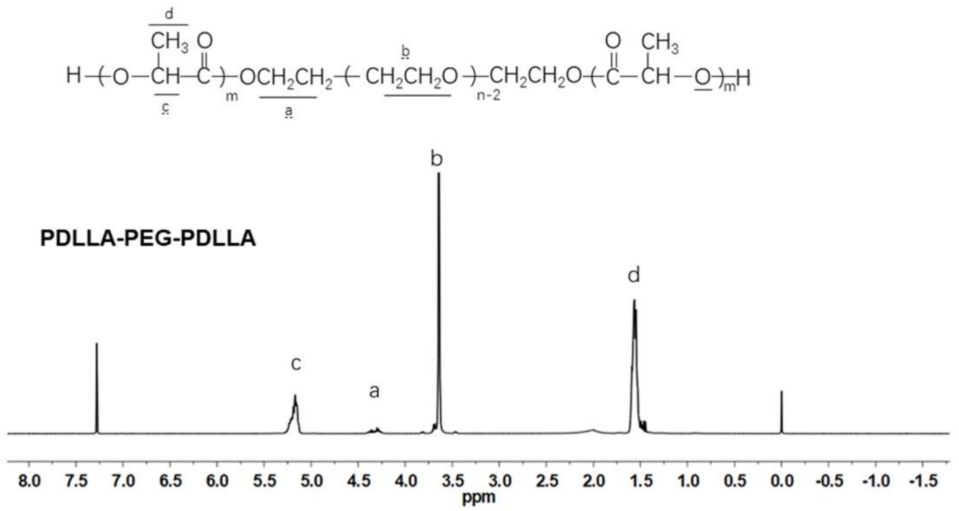


Figure S1. The $^1$H-NMR spectrum of PDLLA-PEG-PDLLA block copolymers in CDCL3.

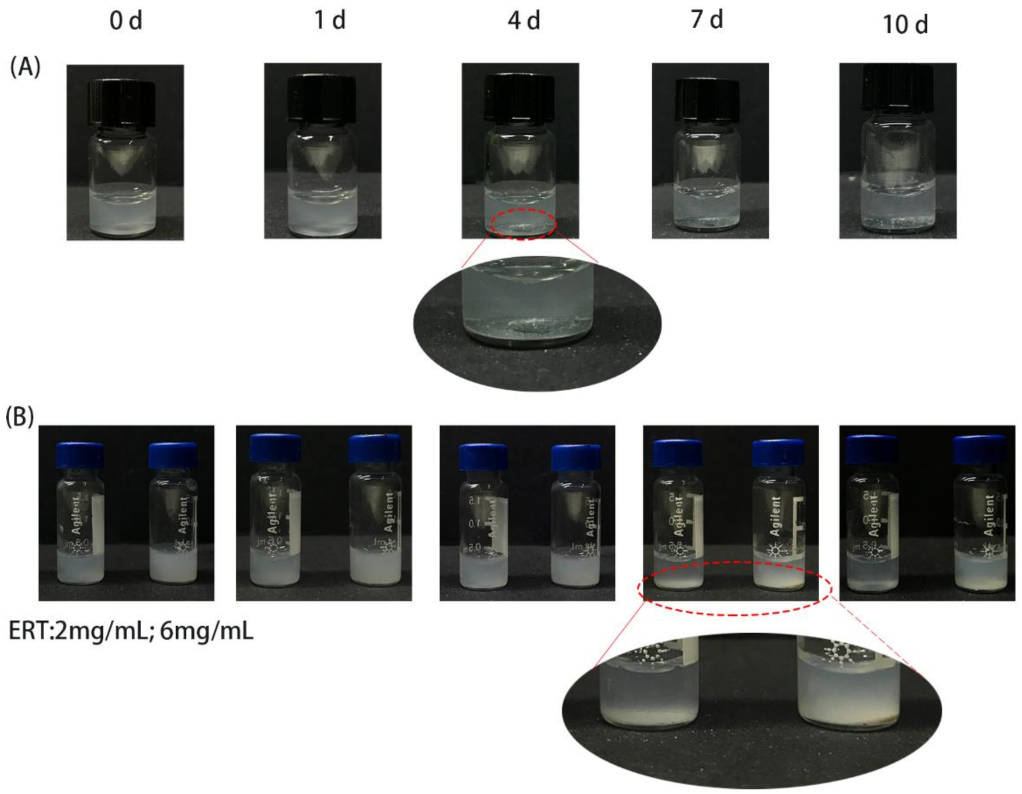


Figure S2. Images of ERT loaded formulations at the indicated time and the images were representative of n = 3 at each time. The hydrophobic ERT powder diffused out of the HMSNs and gel was emphasized by the dashed ellipse in the image.

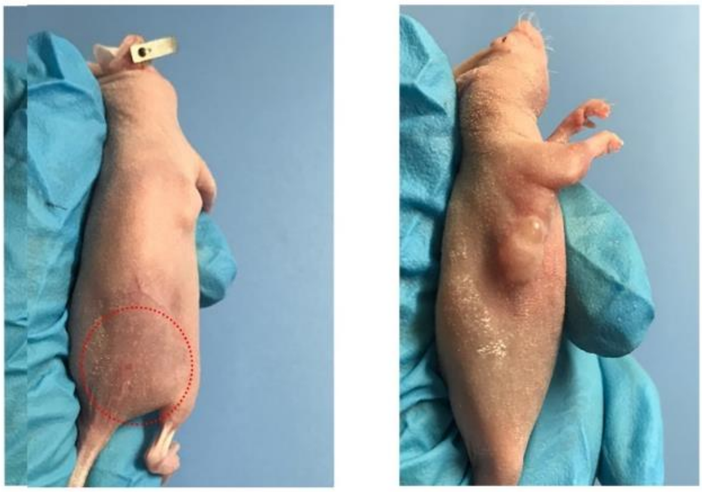

Figure S3. Skin lesions in mice administered high dose Tarceva® (100mg/kg/day).
